# Different Pathways of Skin Aging: Objective Instrumental Evaluation

**DOI:** 10.3390/diagnostics14212381

**Published:** 2024-10-25

**Authors:** Noah Musolff, Carmen Cantisani, Stefania Guida, Simone Michelini, Madeline Tchack, Babar Rao, Giovanni Pellacani

**Affiliations:** 1 Dermatology Clinic, Department of Internal, Anesthesiologic and Cardiovascular Sciences, “Sapienza” University of Rome, 00161 Rome, Italy; 2 Rao Dermatology, New York, NY 10003, USA; mst122@rwjms.rutgers.edu (M.T.); babarrao@gmail.com (B.R.); 3 School of Medicine, Vita-Salute San Raffaele University, 20132 Milan, Italy; 4 Department of Dermatology, Rutgers Robert Wood Johnson Medical School, Clinical Academic Building, 125 Paterson St., New Brunswick, NJ 08901, USA; 5 Rutgers Center for Dermatology, Somerset, NJ 08873, USA; 6 Department of Dermatology, Weill Cornell Medicine, New York, NY 10021, USA

**Keywords:** non-invasive imaging, D-OCT, VISIA, skin aging

## Abstract

**Background/Objectives**: Hypertrophic and atrophic skin aging represent two distinct phenotypes: hypertrophic aging is marked by deep wrinkles and a leathery texture, whereas atrophic aging is characterized by overall skin thinning, increased vascularity, and a higher risk of non-melanoma skin cancers. This study aims to elucidate the characteristics and differences between hypertrophic and atrophic facial aging subtypes using two non-invasive imaging devices: VISIA^®^ and dynamic optical coherence tomography (D-OCT). **Methods**: We retrospectively evaluated patients who had presented to the outpatient dermatological clinic at Policlinico Umberto I hospital in Rome, Italy for a non-invasive facial imaging check-up. We included 40 patients aged 60–75 who were imaged with VISIA^®^ and dynamic optical coherence tomography (D-OCT). Based on the number of UV spots and amount of red found on VISIA^®^, subjects were grouped into four subgroups (PIGM, RED, CONTROL, PIGM + RED), and trends among them were analyzed. **Results**: We found a strong correlation between VISIA^®^ red area scores and D-OCT vascular density at 300 µm depth, confirming VISIA^®^’s effectiveness for assessing facial vascularity. Wrinkle count was highest in areas with UV spots, particularly in the PIGM and PIGM + RED groups. Conversely, low attenuation coefficients and dermal density were observed in regions with low UV spots but high red areas. Intermediate subgroups (CONTROL and PIGM + RED) displayed varying parameters. **Conclusions**: Non-invasive imaging devices are effective in evaluating facial aging and distinguishing between aging subtypes. This study identified two intermediate aging types in addition to the hypertrophic and atrophic subtypes.

## 1. Introduction

As we age, the skin undergoes noticeable changes due to intrinsic and extrinsic aging factors. Intrinsic aging is a natural process determined by genetics, characterized by a gradual decline in biological functions over time leading to the loss of collagen and elastin, proteins that provide skin with high elasticity and firmness, resulting in thinner and more fragile skin. Extrinsic aging, on the other hand, is caused by external factors such as exposure to the sun, pollution, and lifestyle factors such as smoking and diet. Extrinsic aging causes damage to the skin and accelerates the natural aging process [1,2]. Clinically, the combined effects of intrinsic and extrinsic aging manifest in the formation of wrinkles, fine lines, sagging skin, hyper- and hypopigmentation, and an uneven skin tone [3]. 

Evaluating facial skin aging involves various methods, including clinical assessments, scales, imaging techniques, and biopsies. The initial step is typically a clinical examination of the patient’s face, often supported by clinical scales. Several scales have been developed to objectively assess facial skin, such as the Glogau, Fitzpatrick, and Roberts scales. For example, the Glogau scale is a four-point rating used to classify the severity of photodamage from I to IV, ranging from “no wrinkles” to “only wrinkles” [4]. Additionally, new scales are continually being developed and adapted [5]. Despite the wide availability of scales, their use remains subjective, with different institutions and researchers preferring different scales.

Skin aging is a highly complex and non-linear process, influenced by numerous extrinsic and intrinsic factors, leading to a wide variety of manifestations and phenotypes. To simplify this complex process, a recent distinction between hypertrophic and atrophic skin aging has been proposed, describing two phenotypes of aging that differ in skin structure and appearance. Hypertrophic skin aging features deep wrinkles, thick furrows, and a leathery texture, with an increase in tissue in the dermis due to solar elastosis, while the epidermis thins and shows dyspigmentation. Atrophic skin aging involves overall skin thinning, telangiectasias, translucent skin, stellate pseudoscarring, and focal depigmentation. This aging subtype has a higher risk of developing keratinocyte skin cancers, such as basal cell carcinomas (BCC) and squamous cell carcinomas (SCC), potentially due to increased vascularity and/or minimal solar elastosis preventing tumor penetration into deeper layers [6,7,8]. Additionally, the two phenotypes have been found to be associated with biological sex: the atrophic phenotype is more common among men whereas the hypertrophic one tends to develop more in women [9]. However, classifying any subject into one specific category is often very difficult, as alterations typical of both categories are usually present.

The need for standardization in studying skin aging characteristics is increasingly important in the expanding field of dermo-aesthetics and the numerous active ingredients proposed for skin rejuvenation. Standardized digital photographic systems are non-invasive imaging tools that consist of a camera and a set of lights that capture high-resolution digital images of the patient’s face under different lighting conditions, including UV and cross-polarized light. The images can then be analyzed using advanced algorithms and dedicated software, providing a comprehensive assessment of the patient’s skin condition [10,11,12,13]. Non-invasive imaging has recently become a significant tool in dermatology, offering precise ways to study biological structures not visible to the naked eye. Traditional methods such as dermoscopy, along with more advanced techniques like confocal reflectance microscopy, optical coherence tomography (OCT), and high-frequency ultrasound, are now widely used [14,15,16]. OCT is an imaging technique that uses light waves to create high-resolution images of biological tissues, including skin. In the study of aging, OCT has been successfully used to demonstrate differences in the skin of individuals of different ages [17]. It is particularly useful for assessing epidermal thickness, dermal and stroma fiber density, and organization. Dynamic mode OCT (D-OCT) enables the visualization and quantification of the superficial vascular architecture.

Recently, Pezzini et al. conducted a thorough analysis of variations in clinical skin parameters, obtained through standard image digital analysis, and cyto-architectural features, evaluated using reflectance microscopy and optical coherence tomography, according to aging in 20-to-90-year-old subjects [18]. The authors found that the most striking visible changes with aging were related to increased redness and pigmented spots (corresponding to red areas and ultraviolet (UV) spots upon standard image digital analysis), with wrinkles showing an overall increase but with a wide standard deviation [19]. 

This study aims to further elucidate the characteristics and differences of facial aging subtypes using non-invasive imaging devices. Facial skin aging is important from both a cosmetological and clinical perspective, as the incidence of certain skin cancers appears to depend on aging subtypes, and we hope both fields will benefit from our results. VISIA^®’s^ objective parameters were used to categorize subjects according to the predominance of visible aging features, corresponding to skin redness and pigmented spots, and optical coherence tomography was used to define the microscopic substrates in the different groups.

## 2. Materials and Methods

### 2.1. Study Subjects

We retrospectively evaluated patients who had presented in 2023 to the dermatology outpatient clinics at Policlinico Umberto I hospital in Rome, Italy for a non-invasive facial imaging check-up. Prior to imaging, each patient gave their written consent as part of everyday practice, including consent for the use of photographs. The patient and imaging data were extracted from the local database, and we eventually included 40 patients aged 60 to 75 years without any major facial surgical interventions or invasive cosmetic facial procedures in the past. Patients who did not meet these criteria were excluded from the study.

### 2.2. Imaging Check-Up Procedure

The first step involves examining all participants with the VISIA^®^ (7th generation, Canfield Scientific, Inc., Parsippany, NJ, USA) imaging system. VISIA^®^ images are taken frontally and at 33° angles from the left and right sides. The frontal pictures are then analyzed using the intrinsic VISIA^®^ software (version 7.0) for wrinkles, pores, UV spots, brown spots, red areas, and bacterial porphyrins. The absolute feature count is extracted and further analyzed.

As a second step, patients undergo image acquisition with the D-OCT device (VivoSight Dx, Michelson Diagnostics Ltd., Kent, UK) on both the right and left zygomatic arches. The device is set to dynamic mode to include vascular measurements.

Finally, an expert trained in non-invasive imaging modalities evaluates the facial clinical appearance, quantitative VISIA^®^ measurements, and skin morphology on D-OCT scans to generate a final report for the patient.

### 2.3. Study Aims and Protocol

**Figure 1:**
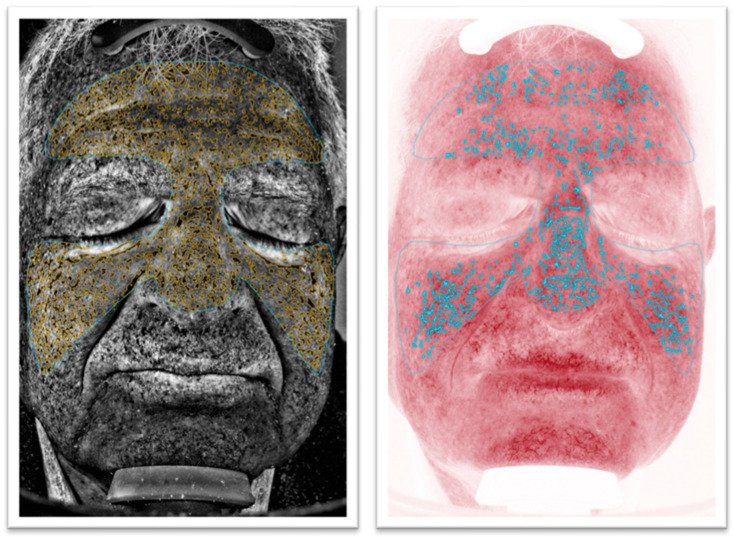
VISIA^®^ images of group RED. On the left the UV spot count is measured, and, on the right, the red areas are measured. The red area count is visibly higher than the UV spot count.

**Figure 2:**
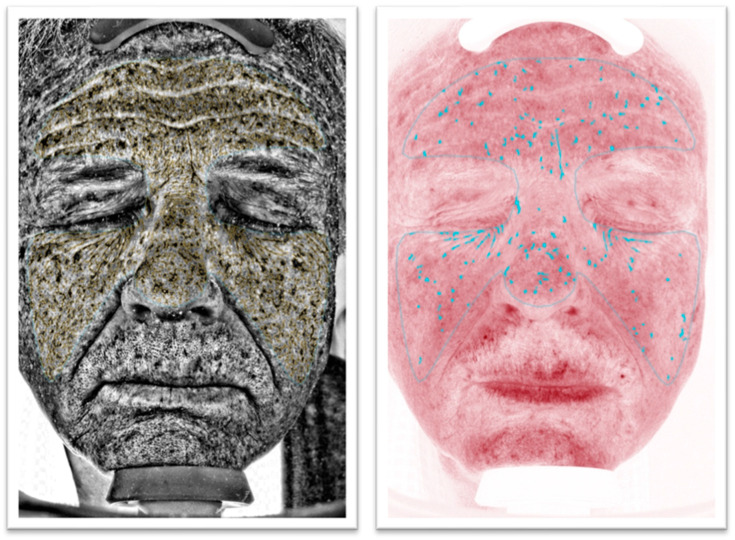
VISIA^®^ images of group UV. On the left the UV spot count is measured, and, on the right, the red areas are measured. The UV count is visibly higher than the red area count.

This study aimed to explore the ability of non-invasive imaging devices to evaluate different aging subtypes. Hence, the protocol was designed to obtain 4 groups of 10 patients based on their VISIA^®^ results for red areas and UV spots extent (Figure 1 and Figure 2):

1. High UV spots and low red areas (Group PIGM [= ”pigmented”]).
2. Low UV spots and high red areas (Group RED).
3. Low UV spots and low red areas (Group CONTROL).
4. High UV spots and high red areas (Group PIGM + RED).

Thresholds were chosen as follows:

- High UV spots ≥ 30,000; low UV spots < 30,000
- High Red areas ≥ 25,000; low red areas < 25,000

### 2.4. Image and Statistical Analysis

**Figure 3:**
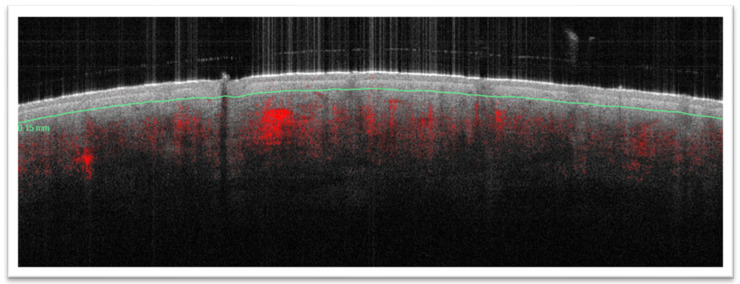
Vertical view of D-OCT scan of zygomatic arch. The epidermis can be clearly demarcated from the dermis which exhibits much vascularity.

**Figure 4:**
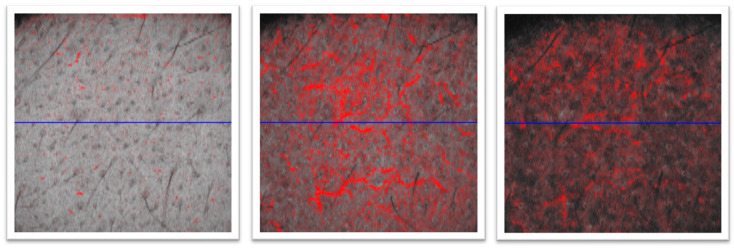
Enface views of D-OCT scan of zygomatic arch. Left: 150 µm. Middle: 300 µm. Right: 500 µm. Vascularity captured by D-OCT is highest at 300 µm.

For this study, the absolute feature counts for wrinkles, pores, UV spots, brown spots, red areas, and bacterial porphyrins were extracted (Figure 1 and Figure 2). Additionally, D-OCT images (Figure 3 and Figure 4) were further analyzed using the VivoTools (V 1.3) software and ImageJ (V 1.53t) for epidermal thickness, attenuation coefficient, dermal density, surface roughness (Ra), and vascular density at 300 µm (VivoTools) and at 150 µm, 300 µm, and 500 µm (ImageJ). All parameters described above were extracted from each frontal VISIA^®^ and both left and right D-OCT scans for each individual. Finally, all collected values were arranged in a correlation matrix using Spearman’s Rho. Trends among the other parameters of VISIA^®^ and D-OCT were then observed and compared within each group, and a *p*-value of <0.05 was considered significant.

## 3. Results

Among the 40 included study subjects, 18 were male and 22 were female. The mean age for males was 68 ± 2.3 years, while for females it was 71 ± 2.1 years. Each of the four subgroups consisted of 10 subjects.

### 3.1. VISIA^®^ Evaluation

UV spots are the result of a quantitative analysis of UV damage seen under polarized light, representative of underlying pigmentation, while red areas in VISIA^®^ refer to specific regions of the skin that exhibit a higher intensity of redness compared to the surrounding areas. Clinically, red areas were found to be linked to areas of erythema and telangiectasias. In the cross-analysis, they were positively correlated to brown spots (*p* = 0.03), pores (*p* = 0.009) and porphyrins (*p* = 0.025). Of interest was also their positive correlation with vascular density (see D-OCT evaluation) at 150 μm and 300 μm (*p* = 0.022 and 0.04, respectively). The presence of pores was positively correlated with wrinkle count (*p* 0.006). Brown spots in VISIA^®^ refer to specific areas of the skin that exhibit a darker pigmentation compared to the surrounding skin. Apart from their association with red areas, they also showed a positive correlation to wrinkles (quantification of fine lines and furrows, *p* = 0.025) and UV spots (*p* = 0.009). By detecting the presence of porphyrins under polarized light, VISIA^®^ estimates the presence of bacteria on the skin’s surface. They were linked to red areas (see above). Furthermore, they had a positive association with pores (*p* = 0.001) and UV spots (*p* = 0.003).

### 3.2. D-OCT Evaluation

Vascularity was assessed with different methods. On VivoTools, the chosen depth was 300 μm to obtain a valid result for the dermis’ vascularity. At this depth, the vascular density as per VivoTools was positively correlated with the measurements obtained with ImageJ at 150 μm (*p* = 0.006), at 300 μm (*p* < 0.0001), and at 500 μm (*p* < 0.0001). Unsurprisingly, these values were also interrelated: 150 μm and 300 μm (*p* < 0.0001), 300 μm and 500 μm (*p* < 0.0001). The vascular density at 300 μm was also positively associated with the epidermal thickness (*p* = 0.004). The D-OCT attenuation coefficient is a measure of how light intensity decreases as it propagates through different layers of tissue during D-OCT imaging. The attenuation coefficient calculated by VivoTools on D-OCT was related to two variables: UV and brown spots. Both appeared to be negatively correlated: with *p*-values of 0.012 and 0.004, respectively. Dermal density refers to the brightness or intensity of the signal returned from the dermis. Denser regions, hypothesized to partly correspond to higher collagen content, reflect more light and appear brighter in the image. In individuals with a decreased dermal density, epidermal thickness increased, and vice versa (*p* = 0.017). Surface roughness (Ra) was not significantly correlated to other variables.

### 3.3. Subgroup Cross-Analysis

After performing descriptive statistical analysis, trends in VISIA^®^ and D-OCT parameters could be observed. As expected, the highest UV count was found to be in groups PIGM and PIGM + RED, while the most red areas were in groups RED and UV + RED.

Among the four subgroups, four significant trends could be observed (Table 1, *p* < 0.05):

1. Wrinkle count: RED < CONTROL < PIGM < PIGM + RED
  a. The wrinkle count was lowest in the groups that had the lowest UV spot count.
2. Attenuation coefficient: PIGM + RED < PIGM < RED < CONTROL
  a. The attenuation coefficient was highest in subjects with a low UV spot count.
3. Dermal density: PIGM + RED < PIGM < CONTROL < RED
  a. The dermal density was highest in subjects with a low UV spot count.
4. Vascular density at 300 µm: CONTROL/PIGM < PIGM + RED < RED
  a. The vascular density was highest in subjects with the highest red area count.

## 4. Discussion

Skin aging is a natural process characterized by the deterioration of cutaneous and subcutaneous tissues, leading to wrinkles, fine lines, sagging, and dyspigmentation. In the dermis, one of the primary changes is the degradation and reduced production of collagen and elastin fibers. While the aesthetic implications of facial skin aging drive a significant industry focused on reversal and prevention, there are also important clinical implications. Keratinocyte skin cancers, such as basal cell carcinoma (BCC) and squamous cell carcinoma (SCC), are common and are linked to the accumulation of mutations in keratinocytes, exacerbated by advanced age and photoaging [19,20,21]. The skin’s diminished ability to repair itself and resist damage, combined with factors like epidermal thinning, decreased sebaceous gland activity, reduced collagen and elastic fibers, and a weakened immune response, may contribute to this susceptibility [22,23]. 

J. Ayer and C. Griffiths first introduced the terms hypertrophic and atrophic skin aging to describe two distinct forms of aging: hypertrophic aging, characterized by pronounced wrinkling and depigmentation, and atrophic aging, marked by minimal wrinkling, skin thinning, and an increased presence of telangiectasias [24]. While their analysis using VISIA^®^ did not reveal significant differences between the two phenotypes, histological evaluation of facial skin samples showed that the atrophic phenotype had a significantly thicker epidermis compared to the hypertrophic phenotype. This difference was further supported by histological samples from the buttocks, where the atrophic phenotype again exhibited a thicker epidermis. Furthermore, individuals with atrophic facial skin demonstrated higher von Willebrand factor (vWF) expression, indicating increased vasculature compared to hypertrophic facial skin, and a significantly greater number of blood vessels compared to hypertrophic skin in both male and female participants. Since then, multiple studies have delved deeper into distinguishing these phenotypes, leading to the development of photonumeric scales [25,26]. Histological analysis has revealed that hypertrophic skin tends to have an increased amount of solar elastosis and a reduced epidermal thickness [7]. Additionally, Collagen VII—a key component at the dermal-epidermal junction, typically reduced in aged skin—appears to decline at an accelerated rate in atrophic skin [7]. Atrophic skin also shows a notable reduction in CD44, a major cell surface receptor for hyaluronic acid [7]. 

Apart from histological differences, large observational studies have noted differences in certain dermatological neoplasms. In studies looking at atrophic skin, a higher prevalence of actinic keratoses (AKs), seborrheic keratoses, and a history of skin cancer has been demonstrated [6,7,27]. Moreover, in 2001, R. C. Brooke and colleagues discovered a discordance between facial wrinkling and the presence of basal cell carcinoma (BCC) [8]. The researchers investigated the relationship between facial wrinkling and BCC occurrence, observing that individuals with BCCs had smoother, less wrinkled facial skin compared to those without skin cancer. The results of the study revealed that, despite being older on average, patients with BCCs had a lower mean grade of facial wrinkling compared to the control group. Through logistic regression analysis and controlling for age, sex, and smoking history, the researchers found that an increase in the grade of facial wrinkling was associated with a progressively reduced likelihood of developing BCCs. The maximum protective effect against BCCs was observed at the highest wrinkling grade [8]. 

The atrophic and hypertrophic phenotypes of skin aging have been primarily defined through clinical examination, later validated by histological findings. However, clinical evaluation can be subjective, and histology is not practical for large-scale studies. This led us to identify the need for a more objective method to assess aging subtypes. Our study aimed to use VISIA^®^ and dynamic optical coherence tomography (D-OCT) to further characterize what has already been described in the literature. Both devices have demonstrated utility in quantifying the effects of aging [28,29]. In this study, VISIA^®^ was employed to capture photographs and analyze the facial aging process of 40 individuals. Subjects were initially classified based on two variables found in the VISIA^®^ analysis: UV spots and red areas. This approach was chosen to reduce reliance on subjective human evaluations, which, even when performed by highly trained professionals, exhibit interrater variability. UV spots are indicative of intense skin damage not visible to the naked eye, caused by melanin accumulation just beneath the skin’s surface (summarized under the label “PIGM”). Meanwhile, red areas are an objective parameter for evaluating vascularity [30,31]. These variables led to the creation of four subgroups: PIGM, RED, CONTROL, and PIGM + RED. Our cross-analysis of several parameters from both VISIA^®^ and D-OCT revealed four key trends (Table 2). First, two separate algorithms used on D-OCT scans to calculate vascular density at a depth of 300 µm showed a strong correlation with the red area score from VISIA^®^, validating VISIA^®^ as a tool for assessing facial vascularity. However, vascularity determined at depths of 150 µm and 500 µm did not significantly correlate with red areas, likely due to generally lower vascular density at shallower depths and the limited penetration ability of D-OCT at deeper depths. Second, wrinkle count was significantly associated with the presence of UV spots, with the PIGM and PIGM + RED groups showing the highest wrinkle counts on VISIA^®^. In contrast, the attenuation coefficient and dermal density, as determined by D-OCT, were highest in areas where UV spots were low and red areas were prominent. We hypothesized that the low attenuation coefficient and dermal density may be associated with elevated levels of solar elastosis. In summary, we demonstrated the value of non-invasive imaging devices in evaluating the facial aging process and identifying at least two distinct aging subtypes. The RED subgroup exhibited increased vascularity, fewer wrinkles, and lower levels of solar elastosis—findings consistent with the atrophic aging type. Conversely, the PIGM group showed opposite trends, aligning with the hypertrophic aging type. However, it is important to acknowledge the presence of intermediate subgroups (CONTROL and PIGM + RED). These subgroups exhibited varying degrees of the above-mentioned parameters, making their impact on clinical aging effects, including the development of skin neoplasms, less predictable.

In conclusion, the atrophic and hypertrophic phenotypes offer a strong theoretical and clinical framework, but a more objective and instrument-based evaluation is necessary to accurately classify and simplify these aging types. We demonstrated the first-ever non-invasive, objective classification of individuals based on their predominant phenotype (i.e., RED face, PIGMENTATION, both, or neither). We believe this approach will aid in enhancing the personalization of cancer prevention and aging treatments. The primary limitation of our study is the small sample size; future research should focus on expanding the population, assessing the significance of intermediate subtypes, and exploring the correlation between skin cancer and each subtype.

## Figures and Tables

**Table 1:** Means calculated for each parameter according to each subgroup. Significant trends are marked in yellow.

	CONTROL (10 Cases), Mean	PIGM (10 Cases), Mean	RED (10 Cases), Mean	PIGM + RED (10 Cases), Mean	*p*-Value
WRINKLE_ABS	25,511.89	26,297.8	13,917.5	28,102.71	0.031
BROWNSPOT_ABS	25,318.22	27,888.8	23,684.62	34,007.29	0.075
REDSPOT_ABS	14,537.89	18,670.6	34,743.88	39,387	<0.001
PORPH_ABS	10,347.67	15,170.5	18,701.25	22,754.43	0.099
PORES_ABS	16,290.78	15,318.8	16,589.75	19,746.71	0.4
UVSPOTS_ABS	23,621.22	38,390.6	25,256.5	43,414	<0.001
Epidermal thickness (µm)	58.41	59.41	58.61	54.85	0.32
Roughness (Ra)	0.01	0.012	0.01	0.001	0.22
Vascular density 300 µm (Vivotools)	0.115	0.136	0.172	0.155	0.003
VASC150 µm (ImageJ)	1789.56	3144	3972.57	4347.86	0.05
VASC300 µm (ImageJ)	15,787.56	14,355.9	38,454.43	23,216.43	0.04
VASC500 µm (ImageJ)	39,380.56	35,394.6	41,036.43	43,642	0.054
Attenuation coefficient	2.36	2.046	2.18	1.9	0.04
Dermal density	0.846	0.834	0.864	0.789	0.032

**Table 2:** Significant trends observed in each subgroup. The groups with the lowest counts are marked with a red arrow, whereas the highest counts are marked with a green arrow.

	Subgroup			
	CONTROL	RED	PIGM	PIGM + RED
**UV spots**	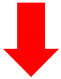	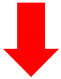	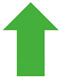	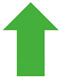
**Red areas**	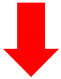	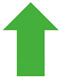	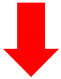	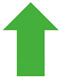
**Wrinkles**	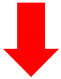	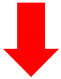	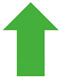	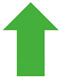
**Attenuation Coefficient**	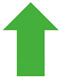	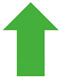	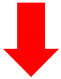	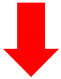
**Dermal Density**	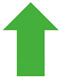	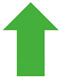	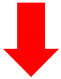	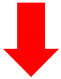
**Vascular Density at 300 µm**	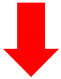	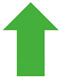	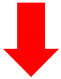	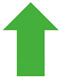

## Data Availability

Dataset available on request from the authors.
